# The Protective Role of Glutathione on Zinc-Induced Neuron Death after Brain Injuries

**DOI:** 10.3390/ijms24032950

**Published:** 2023-02-02

**Authors:** Min Kyu Park, Bo Young Choi, A Ra Kho, Song Hee Lee, Dae Ki Hong, Beom Seok Kang, Si Hyun Lee, Sang Won Suh

**Affiliations:** 1 Department of Physiology, College of Medicine, Hallym University, Chuncheon 24252, Republic of Korea; 2 Institute of Sport Science, Hallym University, Chuncheon 24252, Republic of Korea; 3 Department of Physical Education, Hallym University, Chuncheon 24252, Republic of Korea; 4 Neuroregeneration and Stem Cell Programs, Institute for Cell Engineering, Johns Hopkins University School of Medicine, Baltimore, MD 21205, USA; 5 Department of Neurology, Johns Hopkins University School of Medicine, Baltimore, MD 21205, USA; 6 Department of Pathology and Laboratory Medicine, Emory University School of Medicine, Atlanta, GA 30322, USA

**Keywords:** glutathione, excitatory amino acid carrier 1, zinc transporter 3, zinc, oxidative stress, neuronal death

## Abstract

Glutathione (GSH) is necessary for maintaining physiological antioxidant function, which is responsible for maintaining free radicals derived from reactive oxygen species at low levels and is associated with improved cognitive performance after brain injury. GSH is produced by the linkage of tripeptides that consist of glutamic acid, cysteine, and glycine. The adequate supplementation of GSH has neuroprotective effects in several brain injuries such as cerebral ischemia, hypoglycemia, and traumatic brain injury. Brain injuries produce an excess of reactive oxygen species through complex biochemical cascades, which exacerbates primary neuronal damage. GSH concentrations are known to be closely correlated with the activities of certain genes such as excitatory amino acid carrier 1 (EAAC1), glutamate transporter-associated protein 3–18 (Gtrap3-18), and zinc transporter 3 (ZnT3). Following brain-injury-induced oxidative stress, EAAC1 function is negatively impacted, which then reduces cysteine absorption and impairs neuronal GSH synthesis. In these circumstances, vesicular zinc is also released into the synaptic cleft and then translocated into postsynaptic neurons. The excessive influx of zinc inhibits glutathione reductase, which inhibits GSH’s antioxidant functions in neurons, resulting in neuronal damage and ultimately in the impairment of cognitive function. Therefore, in this review, we explore the overall relationship between zinc and GSH in terms of oxidative stress and neuronal cell death. Furthermore, we seek to understand how the modulation of zinc can rescue brain-insult-induced neuronal death after ischemia, hypoglycemia, and traumatic brain injury.

## 1. Introduction

Glutathione (GSH) is well known as an antioxidant that protects cells from brain-injury-induced reactive oxygen species (ROS) production [1]. GSH is synthesized as a tripeptide consisting of L-cysteine, L-glutamate, and glycine [2]. The concentration of GSH is regulated by glutathione disulfide (GSSG) during the redox cycle [3,4]. GSH peroxidase converts GSH to GSSG with H_2_O_2_, and GSH reductase converts GSSG to GSH with NADPH^+^H^+^ [5]. In various brain injury conditions, the presynaptic release of zinc activates a reaction with free radicals to oxidize the reduced form of GSH to its dimer form (GSSG). Additionally, zinc interferes with the formation of GSH by inhibiting glutathione reductase (GR), an enzyme that converts the dimer GSSG to the GSH form [6]. Consequently, a reduction in GSH concentration contributes to an elevation in intracellular ROS and free zinc levels, leading to the disruption of homeostasis and apoptotic cell death [6].

Zinc has important physiological functions in processes such as protein synthesis, signal transduction, and cell proliferation [7,8]. Under normal conditions, released zinc ionotropically and metabotropically modulates postsynaptic receptors [9,10]. However, under pathological conditions such as stroke, epilepsy, or traumatic brain injury, the excessive influx of zinc into neurons causes neurotoxicity, damage to neurons, and a reduction in GSH [11]. Zinc inhibits glutathione reductase and causes intracellular mitochondrial dysfunction, thereby interfering with GSH synthesis [11,12,13]. Zinc transporter 3 (ZnT3) regulates the homeostasis of zinc and GSH in neurons [12,13]. ZnT3 knockout reduced the translocation of zinc into synaptic vesicles and lowered vesicular zinc concentrations, which enhanced GSH levels through the activation of the GSH synthesis pathway [14,15]. 

GSH is also associated with excitatory amino acid transporters (EAAT1, 2, 3, 4, and 5) [16,17]. EAATs serve a role as the transmembrane complexes that transport glutamate, a major excitatory neurotransmitter, and function as sodium-dependent high-affinity glutamate transporters [16,17]. EAAT1, 2, and 3 are related to the regulation of glutamatergic transmission. EAAT1 is localized in the cerebellum, particularly in astrocytes. EAAT2 is expressed in the forebrain and presynaptic terminals [18,19]. EAAT3 is located in the hippocampus, striatum, and cerebellum [16,17,19]. EAAT4 is primarily present in the cerebellum and is mostly expressed in astrocyte-lineage Bergmann glia and neuronal Purkinje cells. It has a high affinity for the excitatory amino acids L-aspartate and L-glutamate, and previous results suggest that the loss of EAAT4 may contribute to the pathogenesis of spinal cerebellar ataxia type 5 (SCA5) [17,20]. EAAT5 is mainly present in retinal neurons, where it functions to influence light-activated chloride currents mediated by the glutamate activation of EAAT5 [17,20,21]. Among these transporters, mouse EAAT1, which corresponds to human EAAC3, is encoded by the SLC1A1 gene. Mouse EAAT1 has a high affinity with cysteine, which is one of components of GSH [17,19]. Thus, EAAC1 gene-deleted mice show a significant decrease in neural GSH levels with an increase in ROS levels, leading to oxidative damage [22,23]. Previous studies found that cysteine supplementation via N-acetyl-cysteine (NAC) promoted GSH synthesis, eventually exerting a neuroprotective effect. In addition, glutamate transporter-associated protein 3–18 (GTRAP3-18) is known to be a negative regulator of EAAC1 expression [24,25]. Thus, reducing GTRAP3-18 activity enhances GSH synthesis by increasing EAAC1 expression, which modulates the amount of cysteine translocation into the intracellular space [24,25].

In the present review, we show that specific genes such as Gtrap3 and EAAC1 are correlated with the formation of GSH. In the case of brain injury conditions, an excessive amount of free zinc is accumulated in the neuron, which produces ROS via the NADPH oxidase activation pathway. Accumulated zinc also reduces GSH synthesis by the inhibition of glutathione reductase, which aggravates neuron death after stroke, traumatic brain injury, hypoglycemia, and epilepsy. Thus, this review discusses the role of GSH on zinc-induced neuronal death.

## 2. Glutathione Synthesis

Glutathione (GSH) is an antioxidant that protects the brain from the overproduction of reactive oxygen species (ROS), ROS-induced free radical formation, and oxidative stress [26]. GSH consists of L-glutamate, L-cysteine, and glycine, which are linked by adenosine-triphosphate-dependent steps. The GSH formation process is defined as follows [27,28].

**Figure 1:**
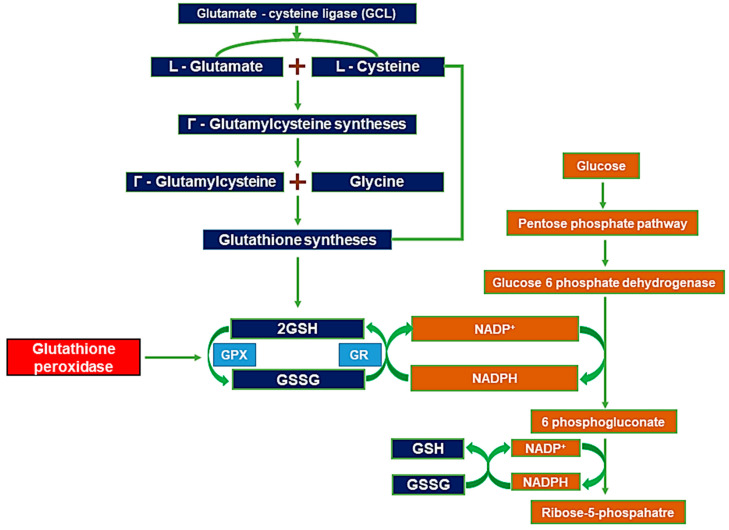
L-glutamate and L-cysteine are bound by glutamate–cysteine ligase enzyme. γ-glutamyl cysteine and glycine combine to form glutathione synthase [31,38,39,40]. GSH and GSSG conversion is mediated by hydroxy peroxide and GSH reductase [41,42,43]. Pentose phosphate pathway (PPP)-derived NADPH supplies electrons to GSSG via GSH reductase [44,45].

L-glutamate and L-cysteine form gamma-glutamylcysteine via the glutamate–cysteine-binding enzyme [29]. Glutamate–cysteine ligase (GCL) catalyzes the synthesis process, which involves ATP-dependent synthesis from L-cysteine and L-glutamate to γ-glutamylcysteine [30]. Glycine binds to γ-glutamyl cysteine to form glutathione synthase for GSH synthesis [31]. GSH has a thiol group (-SH) and its hydrogen provides electrons to remove ROS-induced free radicals. Glutathione exists in its reduced form of GSH (glutathione-SH) and as an oxidized form, GSSG (glutathione-SS-glutathione) [32]. Reduced GSH is oxidized by hydroperoxides and thereby converted to GSSG. Electrons of NADPH are transferred to GSSG by glutathione reductase, which is then transformed to GSH [31,33]. In other words, NADP^+^ is converted to NADPH by glucose-6-phosphate dehydrogenase and the converted NADPH is converted to NADP^+^ by glutathione reductase, which reduces GSSG to GSH [31,33]. GSH is produced by the NADPH present in the cytoplasm through glycolysis [34]. The pentose phosphate pathway (PPP) is a metabolic pathway that oxidizes glucose 6-phosphate (G6P) to pentose phosphate. In addition, astrocytes promote a high influx of glucose into the PPP pathway, which results in the production of ATP and GSH [34,35]. Thus, astrocytes and their normal metabolism play an important role in reducing oxidative stress in the brain and can provide GSH and ATP to neurons by the astrocyte–neuron glutathione shuttle [36,37] (Figure 1).

Glucose-6-phosphate dehydrogenase is involved in the PPP process and converts nicotinamide adenine dinucleotide phosphate (NADP+) to NADPH [46]. Transformed by glucose-6-phosphate dehydrogenase, NADPH delivers high-energy electrons (i.e., reducing equivalents) to cells, maintains cellular redox balance, and supplies reductive biosynthesis [46]. In its reduced form (GSH), it plays a role in removing ROS that cause cellular damage. The sulfhydryl (-SH) moiety, a residue of GSH, neutralizes ROS, and is oxidized to its inactive dimer form (GSSG) when the GSH monomer reacts with ROS [47]. After that, GSH is reduced again by the coenzyme NADPH, formed through a G6PD-6PD-dependent pathway [46,47]. 

Glutathione peroxidase 4 (GPX4) is an enzyme that converts active GSH to GSSG by replacing lipid hydroperoxide (LOOH) with a lipid hydroperoxyl radical (LOO), which is a glucose-6-phosphate dehydrogenase (G6PD) of pentose phosphate. Importantly, glutathione peroxidase 4 (GPX4) converts GSH to GSSG and at the same time converts ROS-induced lipid peroxidase into lipid alcohols, with it being known to play a role in preventing an apoptotic mechanism called ferroptosis [48,49]. The signaling pathway associated with ferroptosis is mainly split into two main pathways: The exogenous, transporter-dependent pathway, which is derived from the amino acid anti-porter system X_c_^−^ inhibition of iron carrier activation, and the intrinsic, enzyme-dependent pathway, which is derived from the reduction of antioxidant enzymes such as GSH or GPX4 [48,49,50]. Finally, the inactivation of GPX4 induced by the GSH depletion caused by multiple neuronal injuries increases intracellular lipid peroxide and induces ferroptosis, which greatly affects neuronal apoptosis [47] (Figure 1). 

Therefore, GSH plays a major role in the suppression of reactive oxygen species, which induce neuronal cell death if unchecked [51]. GSH is synthesized through complex biochemical and cellular mechanisms, and GSH deficiency has been proven to be an important factor that can lead to the upregulation of apoptosis and exacerbate neurological diseases [27,32,33].

## 3. Zinc-Induced Glutathione Deficiency

Zinc is the most abundant metal ion in the brain and is essential for cell growth and proliferation and tissue development [52]. However, the excessive accumulation of zinc in postsynaptic neurons accelerates neuronal injuries [53]. Zinc mediates nicotinamide adenine dinucleotide phosphate oxidase (NOX) activation, which results in intracellular ROS production through an NADPH-oxidase-dependent pathway [54,55]. ROS promotes zinc-induced PARP activation, resulting in ATP depletion and neuronal death [54,55].

**Figure 2:**
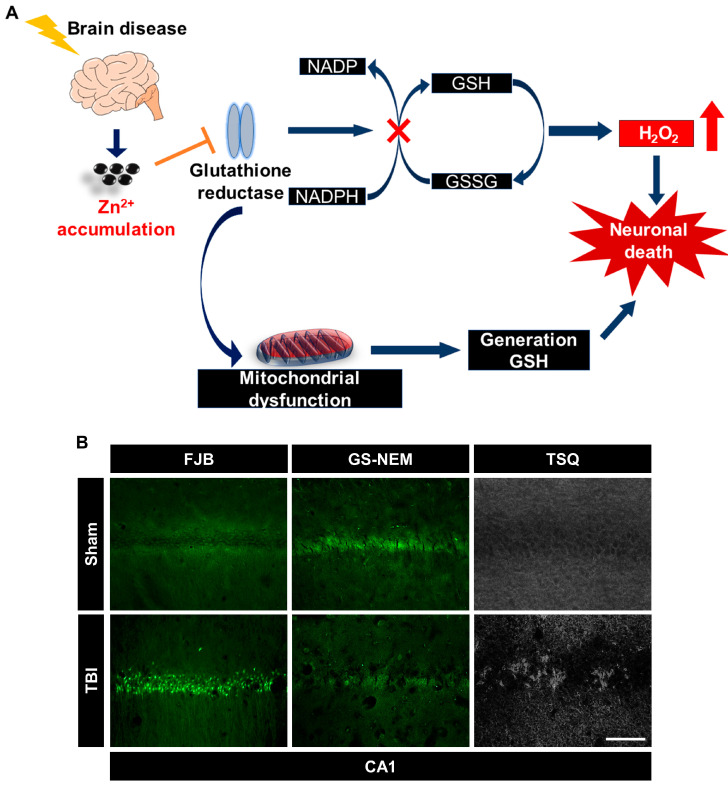
(**A**) Zinc inhibits glutathione reductase (GR) [60]. GR-mediated conversion of GSSG to GSH is inhibited by accumulated zinc [60]. Abnormal GR function leads to mitochondrial dysfunction and disturbs GSH synthesis [59]. Depletion of GSH inhibits GR activity following zinc overload and induces neuronal damage [56]. (**B**) Immunofluorescence images show degenerating neurons in the hippocampal CA1 region 24 h after traumatic brain injury (TBI), detected using Fluoro-Jade B (FJB, green color); immunofluorescence images show degenerating neurons detected using a glutathione antibody, N-ethylmaleimide adduct (GS-NEM, green color), in the hippocampal CA1 region 24 h after TBI; zinc-specific representative fluorescence image displays TSQ (+) neurons by N-(6-methoxy-8-quinolyl)-para-toluene sulfonamide (TSQ) in the hippocampal CA1 region 24 h after TBI. Scale bar = 100 μm.

Zinc accumulation induces oxidative stress by interfering with endogenous antioxidant systems and causing neuronal damage [56,57]. Zinc impairs thiol residue homeostasis, increases lipid peroxidation, and inhibits glutathione reductase to maintain an oxidation-promoting state [56,58]. Astrocytic GSSG was increased under conditions of zinc overload and led to a decreased ratio of GSH transfer to neurons through the astrocyte–neuron glutathione shuttle [36]. In addition, zinc itself makes several contributions to the induction of the lipid peroxidation of sulfhydryl groups in the phosphorus membrane, mitochondrial dysfunction, thioredoxin reductase, and ROS production [58,59]. Consequently, a low concentration of GSH in neurons contributes to oxidative damage after several brain insults [58,59] (Figure 2). 

To enhance neuronal GSH concentrations, several studies have evaluated the chelation of synaptically released zinc using a thiol-containing chemical compound, N-acetyl-cysteine (NAC). The administration of NAC showed a neuroprotective effect after several brain insults [14,15,61]. Brain insults induce the liberation of zinc from zinc-binding proteins such as metallothionein [24,62]. NAC treatment after brain insults revealed that cysteine, an essential component of GSH, supplied the thiol residue of NAC, which has the ability to chelate presynaptically released or detached intraneuronal zinc [24,62]. NAC inhibits transient receptor potential melastatin 2 (TRPM2), one of the nonselective cation channels induced by ROS production, to block the influx of zinc into the cytoplasm of neurons, thereby reducing neuronal cell death and oxidative damage [63]. Moreover, EAAC1 deficiency inhibits the influx of L-cysteine, which reduces the formation of internal GSH [61,62]. Finally, it was found that EAAC1 gene deletion increased basal levels of cytoplasmic free zinc, which later increased neuronal death in hippocampus and cerebral cortex after brain insults [61,62]. 

Based on the above-mentioned results, there is a clear correlation between the amount of zinc and the processes responsible for the synthesis of GSH. The excessive release of zinc from synaptic vesicles or cytoplasmic proteins decreases the synthesis of GSH, which causes neuronal cell death [15,62,63]. Targeting presynaptically released zinc or GSH components via supplementation may have the potential to provide new therapeutic tools to prevent neuronal death after various brain insults [15,62,63].

## 4. EAAC1 and Glutathione Formation

Excitatory amino acid transporters (EAATs, EAACs) regulate neuronal signaling through the influx of glutamate from the synaptic cleft into the neuron. EAATs have five isoforms, of which EAAT1-3 are the most abundantly distributed in the brain [64,65]. EAAT1 (glutamate-aspartate transporter, GLAST) and EAAT2 (glutamate transporter-1, GLT-1) are expressed in neuroglia cells. EAAT3 (exciting amino acid carrier 1, EAAC1) is expressed in mature neurons [64,65,66].

EAAC1 is the primary neuronal glutamate transporter and is responsible for the transport of high-affinity sodium-dependent L-glutamate. EAAC1 also plays an important role in GSH synthesis in the midbrain via the uptake of cysteine into neurons [64,65,66,67,68]. When brain-disease-induced oxidative stress induces EAAC1 dysfunction, caused by peroxynitrite or H_2_O_2_, this reduces the concentration of neuronal cysteine, indicating a disturbance of GSH synthesis in the mouse midbrain [24,63,65,67,68]. The oxidative-stress-induced dysfunction of EAAC1 was further revealed to contribute to neuronal damage in Alzheimer’s and ischemic stroke patients [24,63,65]. In addition, the GTRAP3-18 gene, which is functionally related to the EAAC1 gene, contributes to GSH regulation [25,69,70,71]. GTRAP3-18 is a family of RAB receptors (PRA) that have subtypes of PRA1 and PRA2 [25,69]. The subtypes of PRA1 and PRA2 have been shown to have intracellular positions in the Golgi complex and vesicles (ER) [25]. 

The hydrophobic domain interaction between GTRAP3-18 and EAAC1 inhibits EAAC1 translocation from the ER [25,69]. Thereafter, GTRAP3-18 binds to the C-terminal domain of EAAC1 and inhibits glutamate uptake [25,67,69]. It was also confirmed that EAAC1 was increased in the plasma membrane in GTRAP3-18 gene-deleted mice and the concentrations of cysteine and GSH in the brain were elevated in GTRAP3-18 gene-deleted mice [25,69]. In other words, as GTRAP3-18 gene expression increased, the expression of the EAAC1 gene decreased, and it was confirmed that the two genes play opposite roles. Finally, it was confirmed that the increased expression of GTRAP3-18 caused an increase in oxidative stress, a decrease in GSH levels, and was deleterious to neurons [25,66,69]. 

**Figure 3:**
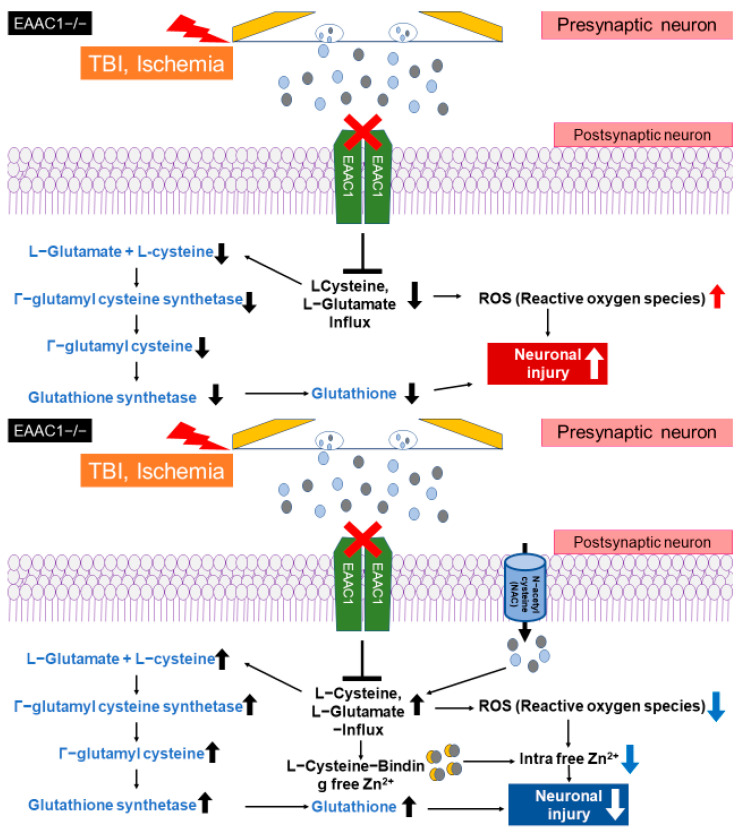
Traumatic brain injury or ischemic stroke when EAAC1 knockout inhibits cysteine translocation [13,73]. Deletion of EAAC1 induced loss of L-cysteine and L-glutamate uptake, and ROS-mediated neuronal damage was preserved [13,73]. NAC can bind to presynaptically released zinc, and cysteine supplementation promotes enhanced GSH synthesis and zinc binding [13,73].

In particular, the EAAC1 gene is highly involved in the entire process of neurogenesis, including cell proliferation, differentiation, and survival after cerebral ischemia [72]. It is important for hippocampal neurogenesis via the regulation of cysteine influx through EAAC1 and its interaction with glutamate [72,73]. EAAC1 deletion promotes increased ischemic damage due to low concentrations of glutathione owing to a lack of cysteine uptake [72,73]. However, when NAC is administered, it acts as a substitute for cysteine, is combined with glutamate, and facilitates GSH synthesis through the GSH synthetic pathway [72,74]. It regulates ROS and glutamate, neurotrophic, and inflammatory pathways via GSH synthesized by NAC and ultimately plays a role in reducing neuronal cell death after cerebral ischemia [63,65,69,72,73,74] (Figure 3). 

## 5. The Role of Glutathione in Zinc-Induced Neuron Death after Brain Injury

### 5.1. Zinc and Glutathione in Stroke 

Global cerebral ischemia (GCI) is caused by a sudden and drastically decreased blood flow to the brain, resulting in primary neuronal damage due to lack of oxygen and energy supply [75]. Secondary damage leads to the disruption of the blood–brain barrier (BBB), activation of microglia, and production of ROS [75]. ROS-mediated free radicals trigger oxidative damage by releasing zinc and glutamate from the presynaptic terminal, leading to the activation of neuronal death cascades [75]. GSH neutralizes free radicals generated through excessive free zinc release after cerebral ischemia and is critical for neuronal survival [75]. Previous studies have demonstrated that EAAC1 gene deletion exacerbates neuronal cell death after cerebral ischemia [13]. In addition, it has been demonstrated that the accumulation of a large amount of zinc after cerebral ischemia is also directly linked to the deletion of the EAAC1 gene [13,72,73,74]. Cysteine uptake via EAAC1 contributes to the production of glutathione (GSH), a potent cellular antioxidant. Furthermore, cysteine, a thiol-containing amino acid, has been demonstrated to have a zinc-chelating effect and, consequently, to protect neurons against brain damage [13,72,73,74]. Restricted neuronal cysteine transport due to the deletion of the EAAC1 gene leads to elevated levels of free zinc in the presynaptic terminal and cytoplasm, suggesting that zinc accumulation exacerbates subsequent neuronal cell death after brain injury. In addition, zinc accumulation in neurons triggers the inhibition of glutathione reductase, ROS production, and increased neuronal death [13,72,73,74]. In the absence of functional EAAC1, the administration of the transmembrane cysteine prodrug N-acetylcysteine (NAC) rescues neuronal cysteine homeostasis, a function normally provided by EAAC1, allowing the free passage of cysteine through the cell membrane of neurons into the cytoplasmic compartment [13,72,73,74]. Taken together, we can infer that cysteine introduced through NAC administration inhibits neuronal free zinc influx, strongly suggesting that it plays a protective role in neurons by the enhancing synthesis of glutathione [13,72,73,74]. Although the impairment of cysteine uptake by EAAC1 gene deletion increases free zinc levels in neurons, we demonstrated that NAC treatment reduced neuronal free zinc levels in EAAC1-knockout mice [13,72,73,74]. Therefore, these results demonstrate the important role of EAAC1 in zinc homeostasis and in bolstering endogenous antioxidant defense mechanisms after acute brain injury [72,73,74]. Finally, after the administration of N-acetylcysteine (NAC, 150 mg/kg, i.p.), a membrane-permeable cysteine prodrug, we observed increased GSH content and reduced BBB destruction, vascular disorganization, and neuronal cell death after GCI [73].

Protocatechuic acid (PCA), one of the major metabolites of antioxidant polyphenols, has a strong antioxidant effect on cells, an antiproliferative effect on tumor cells, and a protective effect against neuronal apoptosis after cerebral ischemia [75]. In particular, PCA has a neuroprotective effect against neuronal cell death through the inhibition of ROS due to the antioxidant effect of PCA [75]. The administration of PCA (30 mg/kg, p.o.) after GCI increased hippocampal glutathione levels and reduced neuronal cell death, ROS production, and BBB disruption. In addition, as an inflammatory mediator, PCA plays a key role in reducing the activity of microglia and astrocytes [75] (Table 1).

### 5.2. Zinc and Glutathione in Traumatic Brain Injury

Traumatic brain injury (TBI) is one of the most prevalent brain disorders and is caused by physical trauma from an accident or violent impact to the head [24,80]. TBI causes edema of the brain as a primary injury and a vast and diverse inflammatory response, followed by secondary damage which leads to neuronal death [24,80,81]. In the secondary damage of TBI, oxidative stress and the activation of microglia and astrocytes play a large role [24,76]. Glutathione deletion and the accumulation of zinc in neurons induces oxidative stress, which promotes neuronal death [24,76]. It is significant that the neuronal death that occurs when a traumatic brain injury is induced in mice deficient in the EAAC1 gene, which is related to deficient glutathione synthesis, can be prevented by a single dose of NAC (150 mg/kg, i.p.), which acts as an alternative source of cysteine in the absence of functional EAAC1 [24,72]. 

In addition, the TBI-induced loss of GSH via the influx of excessive presynaptically released zinc and ROS production was shown to trigger neuronal cell death and cognitive impairment [76]. However, previous studies found that the administration of PCA (30 mg/kg, i.p.), known as a powerful antioxidant, had a neuroprotective effect in the context of TBI by enhancing GSH levels. Moreover, PCA treatment after TBI reduced dendritic damage in the hippocampus and cortex, reduced microglia and macrophage activation, and prevented delayed neuronal death after TBI [76] (Table 1).

### 5.3. Zinc and Glutathione in Hypoglycemia

Hypoglycemic brain damage is caused by insufficient glucose supply, especially in diabetic patients on insulin therapy [78,79]. Hypoglycemia induces low-frequency EEG activity, which can lead to cognitive impairment and neuronal cell death due to low glucose and ATP depletion [78,79]. To recover from the hypoglycemic condition, glucose reperfusion is necessary. However, rapid glucose reperfusion also triggers secondary damage, called “glucose-reperfusion injury”. Glucose reperfusion causes ROS production, the activation of glutamate receptors, and the release of extracellular zinc, triggering neuronal cell death [78,79]. In particular, when zinc influx arises from presynaptic terminals, poly(ADP-ribose)polymerase (PARP) is activated, resulting in ATP depletion, superoxide production, and a lack of GSH [78,79]. However, as a result of administering NAC (300 mg/kg, i.p.) after hypoglycemia, translocated zinc into the hippocampal neurons is chelated and GSH levels are increased, thereby reducing oxidative damage, BBB disruption, microglia activation, and neuronal death [78,79] (Table 1).

Recurrent moderate hypoglycemia (R/M hypoglycemia)-induced PARP-1 activation depends on cytoplasmic NAD. NAD is an essential component of glycolysis, and even when glucose availability is restored by PARP-1 activation after hypoglycemia, cells become unable to use glucose, resulting in ATP depletion [78,79]. The depletion of ATP leads to oxidative damage, zinc accumulation, and the depletion of GSH [78,79]. However, the administration of pyruvate acid (500 mg/kg, i.p.), which provides an alternative source of ATP production through the TCA cycle, has been shown to decrease dendritic damage, microglia activation, vascular loss, and zinc accumulation [78,79] (Table 1).

### 5.4. Zinc and Glutathione in Epilepsy

Epilepsy is caused by the excessive activation of excitatory synaptic neurotransmission, or the imbalanced regulation of inhibitory neurotransmission and oxidative stress caused by an imbalance in free radical and nitrogen production [77]. In particular, epilepsy has been identified to lead to a significant increase in oxidative stress, and it has been found that an alteration in the antioxidant system in epilepsy is therefore of considerable importance [77]. Previous studies verified that PCA (25 mg/kg) treatment after pilocarpine-induced (25 mg/kg) epilepsy reduced oxidative damage, microglia activation, and neuronal death, and prevented a deficiency in GSH [77] (Table 1).

### 5.5. Zinc and Glutathione in Brain Injuries

**Figure 4:**
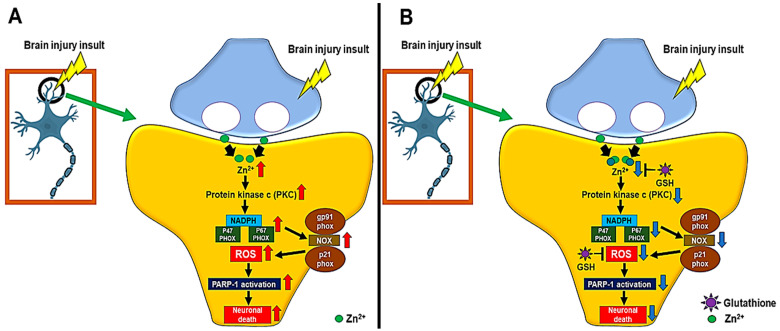
(**A**) When a brain injury occurs, excess vesicular zinc influxes into the cytoplasm of neurons. Then, protein kinase c (PKC) is overexpressed by the vesicular zinc [81,82,83,84]. Due to activated PKC, nicotinamide adenine dinucleotide phosphate (NADPH) is activated, and, as a result, p47(phox) and p67(phox) of NADPH subunits activate gp91(phox) and p21(phox) present in the neuron membrane to generate superoxide [81,82,83,84]. (**B**) However, when GSH is formed, it binds to free zinc to reduce NADPH oxidase activation, which thereby reduces the final product, ROS, or GSH prevents neuronal death by acting as an antioxidant, reducing ROS directly [13,72,73,74].

Taken together, when brain insult occurs, excessive amount of vesicular zinc is released from the presynaptic terminals and translocated into the postsynaptic neurons. Then, accumulated zinc activates PKC (Protein kinase c), which translocates NADPH subunits P47(phox) and P67(phox) to the cytoplasmic membrane [81,82,83,84]. Moved subunits P47(phox) and P67(phox) activate membrane components of gp91 (phox) and p22(phox)) to form superoxide-generating enzyme components [81,82,83,84]. This reaction increases ROS production and increases oxidative stress [78,79,81,82,83,84]. Oxidative stress activates PARP-1 (Poly ADP-Ribose Polimerase-1), which consumes the existing ATP and results in severe neuron damage [78,79,81,82,83,84] (Figure 4A). However, when GSH is present in the postsynaptic neurons, it binds free zinc translocated from the synaptic cleft, thereby reducing the free zinc level, and inhibiting PKC activation [13,72,73,74]. Then zinc-induced PKC activation and ROS production is inhibited [13,72,73,74,77]. Additionally, GSH itself functions as an antioxidant component to reduce ROS, thereby having a protective role against neuronal death after stoke, traumatic brain injury, hypoglycemia and epilepsy [13,72,73,74,77] (Table 1).

## 6. Conclusions

Glutathione (GSH) is a major component of cellular antioxidant systems and has a neuroprotective role in several brain insults. Previous studies have demonstrated that GSH plays a neuroprotective role by inhibiting the generation of reactive oxygen species after cerebral ischemia, traumatic brain injury, hypoglycemia, and epilepsy. The excessive neuronal accumulation of zinc downregulates GSH synthesis, which aggravates neuronal injuries after the above brain insults. Thus, in this review, we discussed evidence that GSH is an essential factor for preventing zinc-induced neuronal death. Furthermore, we suggest that the modulation of zinc in the brain may have neuroprotective effects via the protection or conservation of the integrity of GSH-dependent cellular antioxidant systems.

## Figures and Tables

**Table 1:** Neuroprotective effects of GSH supplementation in several brain insults.

Disorder	Animal Model	Genetic Manipulation	Treatment	Result	References
		Excitatory amino-acid carrier 1 (EAAC1)	Protocatechuic acid (PCA)	ROS ↓	
Stroke	C57BL/6J SD rat	Glutamate transporter-associated protein 3–18 (GTRAP3-18)	N-acetylcysteine (NAC)	activity of microglia and astrocytes ↓ neuronal cell death ↓	[13,22,73,75]
				zinc accumulation ↓ GSH levels ↑	
		^-^		ischemic brain injury ↓	
Traumatic brain injury (TBI)	C57BL/6J SD rat	Excitatory amino-acid carrier 1 (EAAC1) Zinc transporter 3 (ZnT3)	Protocatechuic acid (PCA) N-acetylcysteine (NAC)	ROS ↓ activity of microglia and astrocytes ↓ neuronal cell death ↓ zinc accumulation ↓ traumatic brain injury ↓	[15,24,76]
Epilepsy	SD rat		Protocatechuic acid (PCA)	activity of microglia and astrocytes ↓ neuronal cell death ↓ GSH levels ↑ ROS ↓	[77]
Hypoglycemia	SD rat		Pyruvic acid N-acetylcysteine (NAC)	ROS ↓ activity of microglia and astrocytes ↓ neuronal cell death ↓ zinc accumulation ↓ GSH levels ↑ disruption of the blood–brain barrier (BBB) ↓	[78,79]

## Data Availability

Not applicable.
